# The distance between the religious values of parents and those of children in Israel

**DOI:** 10.3389/fpsyg.2023.939014

**Published:** 2024-02-05

**Authors:** Ela Luria

**Affiliations:** Levinsky College of Education, Bar-Ilan University, Ramat Gan, Israel

**Keywords:** cultural transmission, values, religiosity, secularity, mixed marriage

## Abstract

Prior research into the socialization of religious values between religious parents and their children indicates that, in homogeneous religious family groups, there are similarities between the religious values of religious parents and those of their children. Research further shows that, in religious-secular heterogeneous family groups, there is a significant distance between the religious parent and their teenagers in terms of religious values. The purpose of this study is to explore the transmission of religious values in homogeneous Modern-Orthodox family groups and in heterogeneous Modern-Orthodox-secular family groups in Israel. Results of the study show that religious distance in values is not dependent on the family type (homogeneous/heterogeneous). However, it appears that, when the transmitter of religious values is a religious parent in both homogeneous and heterogeneous religious and religious-secular family groups, there is a distance in the socialization of religious values between religious parent and their children. Moreover, the study proposes that the effect sizes are smaller in the parent-child religious distance of values in homogeneous Modern-Orthodox family groups in comparison to the parent child religious distance of values in heterogeneous Modern-Orthodox-secular family groups. This study provides support for recent research, suggesting that the transmission of religiosity from parents to their children might function as a mechanism of secularization.

## Review of the literature

A fundamental condition for the continuity of a specific culture is the transmission of values from one generation to the next. It is widely accepted that parents play a crucial role in their children's development of social and moral values and, as such, are the primary socializing agents for their children (Flor and Knapp, 2001; Tam and Lee, 2010).

Most of the studies on the transmission of religious values from parents to children focus on the transmission of religious values in homogenous religious households. Many of these studies relied upon single-item measures, used self-reported differences, or only questioned the child or the parent but not both to evaluate the religious distance. This study, however, measures the parent–child religious distance of religious values in homogeneous Modern-Orthodox family groups and in heterogeneous Modern-Orthodox-secular family groups. This article refers to the comparison between parents and children as “religious distance.”

This study is unique as it supports recent research, suggesting that the religious distance between parents and their children is determined by the Modern-Orthodox identity of the transmitter, regardless of the family type they belong to, suggesting that religious parents may find it difficult to encourage religiosity in their children, which might counter their societal norms (Cragun, 2017).

## The differentiation process

Previous research refers to two types of parental values called the differentiation process of values: the first type, known as “parental values,” pertains to the values that parents themselves hold and respect, and the second type, known as “parents' socialization values,” is defined as values that parents wish to transfer to their children that they may not hold for themselves (Knafo and Galansky, 2008; Tam and Lee, 2010).

The values of parents and parents' socialization of values positively correlate; however, the correlations are moderate (Tam and Lee, 2010), that is, parents' socialization of values and parental values are sometimes observed by the parents as two different sets of values (Benish-Weisman et al., 2013). Therefore, these terms are not always congruent. “Sometimes parents see the need to differentiate between their personal values and what they want for their children. Parents understand that they need to prepare their children for social life as it exists in the present and future” (Tam and Lee, 2010, p. 175). According to Schwartz, values form a quasi-circumplex structure, representing conflict and capabilities (Schwartz, 1992).

According to Schwartz (2012), in the socialization process, some values are found to be more important to adolescents than others. Moreover, self-oriented values (self-direction, stimulation, hedonism, power, and achievement) were found to be more important to adolescents than other-oriented values (benevolence, conformity, tradition, and security). Previous research has shown a correlation between other-oriented values and religiosity. As such, it appears that religious parents find it of the highest priority to instill such values in their children for fear of postmodernism. In other words, Modern-Orthodox parents understand the threat of secularism to their child's religious values, and therefore, they aim to transmit high religious values that will be able to counteract the in-group secular values.

## Religiosity and values

Schwartz, a social psychologist and cross-cultural researcher, is one of the leading researchers on the transmission of values. The Schwartz (1992, 2006) Theory of Basic Human Values, which is based on the Rokeach (1973) Value Survey, illustrates a connection between different types of values. The connection between the different values makes up a general class of values that is different from another class of values. Schwartz classified the values by building 10 general types of values, where each type has several values that are closely related to other values belonging to the same group. Schwartz further divided the 10 value types into four dimensions: openness to change vs. conservation and self-transcendence vs. self-enhancement.

The different dimensions of value types placed on opposite axes hint at the difference between religious and secular groups. Openness to Change and Self-Enhancement were found to be positively correlated with the values of secularity, whereas Conservation and Self-Transcendence were found to be more positively related to values of religiosity (Hofmann-Towfigh, 2007; Saroglou and Munoz-Garcia, 2008).

The Schwartz theory of values was and is used as a framework by researchers to investigate different topics related to values, one of them being religiosity. A previous study investigated the religious distance of religious values from Orthodox parents to children in religiously homogeneous and religious-secularly heterogeneous family groups in Israel using the Schwartz (1992, 2006).

Even though there is variation in the Jewish population with respect to religion within broadly defined groups, distinctions in research are usually made among the following important groups (Okun and Nimrod, 2017): In contemporary Israel, there are five dominant groups with respect to religion; Secular Jews, Traditional Jews, Modern-Orthodox Jews, Ultra-Orthodox Jews, and Muslims (Wald and Shye, 1995; Lazar et al., 2002; Even-Zohar, 2015).

This article refers to Modern-Orthodox Jews, who advocate strict observance of the traditional Jewish law, as opposed to Traditional Jews, a diverse group encompassing a large middle ground between the Modern-Orthodox group and Secular Jews. Ultra-Orthodox Jews are characterized by a narrower and more doctrinal adherence to Jewish law and values than Modern-Orthodox Jews.

## Transmission and gender

In parent–child dyads, Knafo and Schwartz (2003) have further shown that there is a preference for accepting values according to same-sex dyads. In other words, when values are not gender-neutral, sons prefer to accept their father's values and daughters embrace their mother's values (Cunningham, 2001; Kapinus, 2004). Clark et al. (1988) bolstered this claim by arguing that “one would expect gender of parent and child to interact” (p. 464).

On the whole, many research studies (Aldous and Hill, 1965; Axinn and Thornton, 1993; Knafo and Schwartz, 2003; O'Bryan et al., 2004; Gniewosz and Noack, 2012) are consistent with the fact that the relationship between parents and children of the same gender is a prominent variable in the study of transmission of cultural values, especially when addressing the continuation and transmission of religious values from parents to their children.

Hayes and Pittelkow (1993) argued that, when studying the opposite gender of parents and children, sons who have strict mothers as role models and who are educated by disciplinary supervision are more likely to absorb their mothers' beliefs. However, fathers have a greater effect on their daughters when using moral supervision. Kohn et al. (1986) discovered in their study on the transmission of values from parents to children that “apparently, the transmission of values from mother to child requires that children accurately perceive their mothers' values; but the transmission of paternal values can occur even when children misperceive what values their fathers really hold” (p. 88).

Many studies that have examined different cultures and ethnicities have shown that gender contributes to the socialization process (Phalet and Schonpflug, 2001; Carlson and Knoester, 2011). Further studies that examine religion and the transmission of values have accordingly inserted gender into their transmission model as a causal variable in the transmission process. Hayes and Pittelkow (1993) mentioned a disagreement between studies as to whether the father or mother is more influential in the transmission of religious values. However, they concluded that no matter who is more dominant in the process, parents function differently in the transmission of values to their children because of the gender difference between fathers and mothers.

### The transmission of religious values by religious-secular heterogeneous family groups

Previous studies on the transmission of values have shown that the parent–child relationship, namely agreement or disagreement over values between parents, influences the transmission process. According to Nelson and Otnes (2002), conflict over values in a marriage is usually caused by a disagreement over the importance of these values. Owing to the greater tendency for value differences to occur in cross-cultural weddings, distance in values between parents and children and confusion over values are intensified in such situations.

Popenoe (1993), a critic of cultural socialization theory, has argued that marital conflict creates an obstacle in the process of intergenerational transmission of culture from parents to children. When children perceive that their parents have different value systems, they are less ready to accept their parents' value priorities, which can lead to a greater religious distance in religious values (Barni et al., 2011). Bengton et al. argued that the situation of mixed faiths in families considerably complicates the passing on of religiosity to younger generations and, as such, leads to a religious distance between the religious values of religious parents and their children (2009).

### The transmission of values in homogeneous family groups

Different studies have shown that, in the transmission of religious values in homogeneous religious family groups, religious parents influence their children's religiosity (Myers, 2004; Bader and Desmond, 2006; Copen and Silverstein, 2008; Min et al., 2012). These studies argue that the homogeneity of transmitters with reference to the transmitted contents assures the greatest transmission of values (Schonpflug, 2001). “When parents are in agreement, their value messages are more likely to be clear and coherent rather than confusing and contradictory” (Knafo and Schwartz, 2003, p. 593). The transmission of values is more effective when parents share the same cultural trait to transmit to their children (Bisin and Verdier, 2010, p. 12).

### Religious distance in religious values between the religious parent and child

Recent research, however, on the transmission of religiosity in religious homogeneous family groups proposes that, even though religious parents in homogeneous households have greater success in transmitting religiosity to their children in comparison to religious parents in heterogeneous marriages, there is still a significant distance between the religiosity of the religious parents and that of their children (Ozorak, 1989; Cragun et al., 2018). Current secularization trends might harm the similarity in religious values between parents and children (Crockett and Voas, 2006; Cragun, 2017).

These studies imply that the religious distance between religious parents and children in religious values has increased, regardless of the type of family in which the child grows up. Modernism and postmodernism might cause religious distance in religious values (Glenn and Kramer, 1987; Schwadel, 2010) between religious parents and children in both homogeneous religious family groups and heterogeneous religious-secular family groups.

## Religious identity in the modern and postmodern era

Contemporary research asserts that modernism and postmodernism appear to cause religious identity formation crises. Modernism and postmodernism affect different cultures and groups in different ways. Modernism is no longer considered a unified concept that affects all individuals in the same way. However, in contemporary times, modernism is observed on multiple levels, depending on both the modern world that one lives in and the values and lifestyle that one chooses to live by Ben-Rafael and Ben-Chaim (2006) and Cohen-Malayev (2008). “*Multiple modernities* is a concept of cultural diversity or multiplicity that disputes a universal approach to modernity biased by Western experience” (Lee, 2006, p. 358). Multiple modernities are molded from the modern universal, Jewish, and religious aspects (Ben-Rafael and Ben-Chaim, 2006).

Having grown up in a modern and postmodern era, those in emerging adulthood (Arnett, 2000), educated by one or more religious parents, are exposed and susceptible to the universal values and lifestyles of the modern and postmodern world that contrast with the values and lifestyles that they have acquired at home and, as such, cause a religious identity crisis in adolescents (Cohen-Malayev, 2008; Steinberg, 2008; Frisch-Atias, 2012).

When attempting to pass down religious values to their child in the emerging adulthood stage of development, religious parents produce, knowingly or unknowingly, a conflict in their child's values, i.e., between the values that the modern and postmodern world has to offer and the religious values that their religious parents wish to instill in them (Ben-Rafael and Ben-Chaim, 2006; Cohen-Malayev, 2008). As such, the strong emphasis placed by religious parents on transmitting their religious values to their children is in direct opposition to the universal levels of modernity. As a result, the actual transmission of religious values tends to be lower than the desired level envisioned by religious parents for their children.

### The present study

The present study uses the Schwartz theory of values to determine the religious distance in values between Modern-Orthodox parents and their children among three types of family groups in Israel: The first family group consists of a Modern-Orthodox father, mother, and child; the second group consists of a Modern-Orthodox father and a secular mother and child; and the third group consists of a Modern-Orthodox mother and a secular father and child.

The values of the parents in this study are measured as two different variables: Parents' personal values (the values that parents themselves personally hold and respect) and “parents' socialization values” (the values that parents wish to transfer to their children) (Knafo and Galansky, 2008; Tam et al., 2012).

Unlike other studies, this study compares parent(s)-child religious distance of religious values in homogeneous Modern-Orthodox groups and Modern-Orthodox-secular heterogeneous groups. Based on the literature review above, it seems likely that, in heterogeneous Modern-Orthodox-secular family groups, there will be a religious distance in religious values between the Modern-Orthodox parents' values and those of their children.

Furthermore, in accordance with the current literature on the topic of parent–child religious distance, it is hypothesized that the religious distance in religious values will also be found in the intergenerational value transmission of homogeneous Modern-Orthodox family groups due to trends in secularity and postmodernism.

However, in line with previous research that argues that “the transmission of values is more effective when the parents share the cultural trait to socialize their children to *[sic]*” (Bisin and Verdier, 2010, p. 12), smaller effect sizes are expected in the parent–child religious distance of values in homogeneous Modern-Orthodox family groups in comparison to larger effect sizes in the religious distance of heterogeneous Modern-Orthodox-secular family groups. Furthermore, this study seeks to discover whether parent–child religious distance varies between boys and girls.

Therefore, the following hypotheses are proposed:

A. Adolescent children will be statistically significantly less religious than their religious parents in both homogeneous Modern-Orthodox and heterogeneous Modern-Orthodox-secular family groups.
B. Parent–child religious distance in religious values will be greater in the value transmission of heterogeneous Modern-Orthodox-secular family groups than homogeneous Modern-Orthodox family groups. As such, the effect sizes of statistically significant results will be greater in the parent–child religious distance of religious values in heterogeneous Modern-Orthodox family groups compared to homogeneous Modern-Orthodox family groups.
C. Parent–child religious distance will not vary for boys or girls owing to the modern and postmodern eras, where, according to research results, religious parents aim to instill high religious values in their children, regardless of the child's gender.

To test such an approach, the researchers used a repeated measure design: a 3 between-group by 1 within-religious value MANOVA to investigate the measured congruency between parents' socialization of religious values and child's own religious values in Modern-Orthodox-secular mixed family groups and homogeneous Modern-Orthodox family groups. The study further tested the effect sizes of statistically significant differences in parent–child religious distance in religious values.

## Method

### Participants

The participants in the research were core members of 142 family groups in Israel. Each family group consisted of three members of the family: the father, the mother, and an unwed young adult (18-25). The young adult was randomly selected from families with more than one adult child. The study consisted of three different types of family groups: the homogenous Modern-orthodox family group, the mixed “Modern-Orthodox father secular mother” family group, and the mixed “Modern-Orthodox mother-secular father” family group. The subjects were located through advertisements around university campuses, in different religious/secular communities around Israel, and on various websites that focus on religious/secular couples. The study used the probability sampling method by randomly selecting the participants from the pool of participants.

The three groups were defined by a median split of the means of their religious/secular scores using the religiosity scale (SRQ). The homogeneous Modern-Orthodox family group consisted of 73 family units, the “Modern-Orthodox father-secular mother” family group consisted of 32 family units, and the “Modern-Orthodox mother-secular father” family group consisted of 37 family units.

This research was limited to Modern-Orthodox Jews to examine the distance between parents' socialization of religious values and children's religious values. Traditional or Ultra-Orthodox Jews were not included in the study since heterogeneous families that consist of Ultra-Orthodox Jews are rare in Israel. In addition, adding traditional couples to the study might lead to confounding variables.

The demographic characteristics of the sample are presented in Tables 1–3.

**Table 1:** Distribution N, (%) of parent-subjects, according to personal characteristics.

**Characteristics**	**Type**	**Modern-Orthodox family group**	**Modern-Orthodox father secular mother**	**Modern-Orthodox mother secular father**
Parents: Gender	Men and women	73 (35%)	32 (15%)	37 (18%)
Fathers: age	Age: 30–40	1 (1%)	1 (3%)	0
	Age: 40–50	14 (19%)	2 (6%)	3 (8%)
	Age: 50–60	58 (80%)	29 (91%)	34 (92%)
Mothers: age	Age: 30–40	2 (3%)	0	1 (3%)
	Age: 40–50	26 (36%)	12 (38%)	7 (19%)
	Age: 50–60	45 (62%)	20 (63%)	29 (78%)
Fathers: Type of school	School: Religious	58 (80%)	26 (81%)	0
	School: Secular	2 (3%)	3 (9%)	33 (89%)
	School: Mixed	13 (18%)	3 (9%)	4 (11%)
Mothers: Type of school	School: Religious	59 (81%)	0	27 (73%)
	School: Secular	3 (3%)	27 (84%)	2 (5%)
	School: Mixed	11 (15%)	5 (16%)	8 (22%)

**Table 2:** Distribution N, (%) of children-subjects according to personal characteristics.

**Characteristics**	**Type**	**Modern-Orthodox family group**	**Modern-Orthodox father secular mother**	**Modern-Orthodox mother secular father**
Children: Gender	Men	32 (43.8%)	11 (34.4%)	19 (51.4%)
	Women	41 (56.2%)	21 (65.6%)	18 (48.6%)
Children: Age	Age: 18–20	12 (16.4%)	5 (15.6%)	6 (16.2%)
	Age: 20–25	61 (83.6%)	27 (84.4%)	31 (83.8%)
Children: Place of residence	Parents' house	37 (50.7%)	17 (53.1%)	19 (51.4%)
	Roommates	29 (39.7%)	9 (28.1%)	13 (35.1%)
	Alone	7 (9.6%)	6 (18.8%)	5 (13.5%)
Children: Type of school	School: Religious	51 (69.9%)	8 (25%)	8 (21.6%)
	School: Secular	0	12 (37.5%)	14 (37.8%)
	School: mixed	22 (30.1%)	12 (37.5%)	15 (40.5%)

**Table 3:** Means and std. deviations of the number of children and years of marriage.

**Characteristics**	**Family**	**N**	**Minimum**	**Maximum**	**Mean**	**S.D**.
Number of Children	Modern-Orthodox father and mother	73	1	8	4.3	1.3
	Modern-Orthodox father Secular mother	32	1	8	3.5	1.5
	Modern-Orthodox mother Secular father	37	2	7	3.6	1.3
Years of Marriage	Modern-Orthodox father and mother	73	10	40	28.9	5.8
	Modern-Orthodox father Secular mother	32	11	40	28.1	7.5
	Modern-Orthodox mother Secular father	37	10	45	28.6	7.8

### Procedure

The subjects were recruited through advertisements around university campuses (88%), advertisements in different religious/secular communities around Israel (8%), and advertisements on various websites that focus on religious-secular couples (4%) to represent different regions in the country. All the subjects were of similar ethnic backgrounds to rule out differences in cultural groups.

Most of the participants were located through the same channel, namely advertisements in different universities around Israel (Tel Aviv University, Ben Gurion University, Bar-Ilan University, Hebrew University, and Ariel University). The sample is a selective sample, which may have caused those with certain characteristics to respond to the advertisement and participate in the study. However, it is assumed to be one that can be cautiously generalized to the academically educated population that accounts for 49.90% of the total adult Israeli population (World Population Review, 2020).

The participants' religiosity was measured using the Student Religiosity Questionnaire (SRQ) and a Sociodemographic Questionnaire. The research followed all APA ethical guidelines, including IRB approval. Subjects showed interest in the study by calling the number listed in the research advertisement. The study's researcher then interviewed subjects to observe whether they were suitable to participate in the study. After obtaining agreement to participate in the study of values from the researcher, an online demographic questionnaire and a PVQ questionnaire were sent to both the parents and the young adult child via a questionnaire database. The participants were offered an incentive of 50 ILS per family for participating in the study.

The questionnaires were returned via the questionnaire database after signing a consent form to complete each survey independently. The researcher was informed of their return by mail. The questionnaires were then assessed, and the results were analyzed using MANOVA repeated measure analysis. Rather than multi-level modeling, this statistical procedure was used for statistical analysis as our research model does not have the nesting structure necessary for the multi-level modeling procedure. The MANOVA procedure was used to measure the exact differences in religious values between the father, mother, and child in the three family groups.

## Research measures

### Sociodemographic questionnaire

Parents and young adults responded to a sociodemographic questionnaire to ascertain their religious beliefs and other characteristics. The sociodemographic questionnaire included questions about the gender and age of the participant, self-defining questions about the level of one's religiosity and type of family, questions about the background (religious, secular, or mixed) of the participant, and questions about the education framework (religious, secular, or mixed) of the participant. The family groups were defined by a sociodemographic variable that includes the three family groups. Religiosity scales further supported the sociodemographic variable, with secular individuals scoring below 3 on the scale, while religious individuals scored above 3, indicating a higher level of religiosity.

### Student religiosity questionnaire

Following the sociodemographic questionnaire, the Student Religiosity Questionnaire (SRQ) was distributed to measure the participants' religiosity. This 20-item questionnaire was developed by Katz and Schmida (1992) to examine two factors of religiosity, namely religious practices and religious principles. The questionnaire was also used to measure adults' religiosity.

The questionnaire consists of 20 items measured on a five-point scale (1=minimal agreement and 5- maximal agreement), with 10 items measuring religious practices and 10 items measuring religious principles. The first factor, focusing on religious practices, includes issues such as Sabbath observance, inter-sex socializing, dietary law observance, observance of days of mourning, observance of fast days, grace before meals, Shabbat termination prayers, and giving of tithes (a portion of your income). The second factor focuses on religious principles and includes issues such as biblical miracles, rabbinical authority, reward and punishment, individual supervision by God, the resurrection of the dead, creation ex nihilo, oral law, the messianic era, divine law, and prophecy.

The alpha coefficient for religious practices was 0.83, and the alpha coefficient for religious principles was 0.90. The questionnaire was modified by adding five extra questions that measured religious practices to focus on additional items that emphasize the different levels of religiosity between parents and children.

In the current study, validity and reliability were found to be significantly high. The internal consistency of religious practices was α = 0.965 for the father's religious practices, α = 0.968 for the mother's religious practices, and α = 0.963 for the child's religious practices. The internal consistency of religious principles was α = 0.974 for the father's religious principles, α = 0.979 for the mother's religious principles, and α = 0.976 for the child's religious principles.

### Portrait values questionnaire

Following the sociodemographic questionnaire and the Student Religiosity Questionnaire, parents and young adults responded to the PVQ questionnaire that was developed by Schwartz (2006) and designed to examine individuals' religious vs. secular values (Fontaine et al., 2005; Roccas, 2005).

The PVQ questionnaire consists of 40 items measuring 10 different value types, including short verbal portraits of a person's goals, aspirations, and wishes. The short verbal portraits refer implicitly to the ten value types, six of which pertain to secular values: self-direction, stimulation, achievement, hedonism, universalism, and power, and four of which pertain to religious values: tradition, security, conformity, and benevolence (Saroglou et al., 2004; Fontaine et al., 2005; Roccas, 2005).

This research study is based on the questionnaire's values related to religiosity. The religious items, which measured four group types tradition, security, conformity, and benevolence were computed into one measure called religiosity. The measure of religiosity consisted of variables that were repeatedly found to be positively correlated with religion, namely, tradition, security, conformity, and benevolence (Schwartz and Huismans, 1995; Saroglou et al., 2004; Fontaine et al., 2005; Roccas, 2005).

Both parents and children completed the PVQ questionnaire. The parents were asked to answer questions about the values they attempted to inculcate and instill in their children, whereas the children were asked to respond about their own values. This research is based only on the questions that were related to the values of tradition, security, conformity, and benevolence.

Three scales were used to measure the similarities/differences in values between parents and children. Two scales were used to measure parents' values (father/mother) and parents' personal religious values vs. parents' socialization of religious values, and one scale was used to measure child socialization of religious values. The similarities/differences in religious values between parents and children were measured as the gap between the parents' socialization of religious values and the child's own values. Religious distance between parents and children was calculated as an average of the parent's religious values compared to the average of the child's personal values.

The Cronbach coefficient alpha values for each of the three PVQ scales are as follows: α = 0.797 for the mother's socialization of religious values, α = 0.823 for the father's socialization of religious values, and α = 0.710 for the child's religious values.

The Cronbach coefficient alpha values for each of the three PVQ scales in the three family groups are as follows:

Homogeneous religious family group: α = 0.76 for the mother's socialization of religious values, α = 0.7 for the father's socialization of religious values, and α = 0.71 for the child's religious values.

Mixed religious father secular mother family group: α = 0.66 mother's socialization of religious values, α = 0.72 for the father's socialization of religious values, and α = 0.62 for the child's religious values.

Mixed religious mother secular father family group: α = 0.79 for the mother's socialization of religious values, α = 0.8 for the father's socialization of religious values, and α = 0.7 for the child's religious values.

Tables 4, 5 present the internal consistency results of each of the ten different value types for father, mother, and child.

**Table 4:** Internal consistency (**α** Cronbach) of the portrait values questionnaire (PVQ) of the current study.

**Type of values and number of questions**	**Father**	**Mother**	**Child**
1. Conformity (7, 16, 28, 36)	0.75	0.78	0.72
2. Tradition (9, 20, 25, 38)	0.76	0.72	0.63
3. Benevolence (12, 18, 27, 33)	0.81	0.79	0.67
4. Universalism (3, 8, 19, 23, 29, 40)	0.78	0.76	0.71
5. Self-direction (1, 11, 22, 34)	0.80	0.75	0.7
6. Stimulation (6, 15, 30)	0.67	0.69	0.75
7. Hedonism (10, 26, 37)	0.73	0.81	0.77
8. Achievement (4, 13, 24, 32)	0.84	0.82	0.84
9. Power (2, 17, 39)	0.71	0.76	0.64
10. Security (5, 14, 21, 31, 35)	0.73	0.73	0.65

**Table 5:** Internal consistency (**α** Cronbach) of the portrait values questionnaire (PVQ) of the current study of the three family groups.

**Type of values and number of questions**	**Father**	**Mother**	**Child**
**Homogeneous “modern-orthodox” family group**			
1. Conformity (7, 16, 28, 36)	0.63	0.61	0.69
2. Tradition (9, 20, 25, 38)	0.6	0.72	0.57
3. Benevolence (12, 18, 27, 33)	0.72	0.82	0.71
4. Universalism (3, 8, 19, 23, 29, 40)	0.66	0.74	0.66
5. Self-direction (1, 11, 22, 34)	0.79	0.74	0.65
6. Stimulation (6, 15, 30)	0.65	0.74	0.7
7. Hedonism (10, 26, 37)	0.72	0.78	0.78
8. Achievement (4, 13, 24, 32)	0.86	0.8	0.82
9. Power (2, 17, 39)	0.72	0.75	0.66
10. Security (5, 14, 21, 31, 35)	0.66	0.66	0.66
**Heterogeneous “modern-orthodox father secular mother”** **family group**			
1. Conformity (7, 16, 28, 36)	0.74	0.77	0.64
2. Tradition (9, 20, 25, 38)	0.75	0.67	0.55
3. Benevolence (12, 18, 27, 33)	0.88	0.52	0.71
4. Universalism (3, 8, 19, 23, 29, 40)	0.77	0.61	0.81
5. Self-direction (1, 11, 22, 34)	0.62	0.59	0.77
6. Stimulation (6, 15, 30)	0.67	0.65	0.8
7. Hedonism (10, 26, 37)	0.63	0.77	0.81
8. Achievement (4, 13, 24, 32)	0.81	0.81	0.81
9. Power (2, 17, 39)	0.76	0.68	0.52
10. Security (5, 14, 21, 31, 35)	0.61	0.59	0.59
**Heterogeneous “Modern-orthodox mother secular father”** **family group**			
1. Conformity (7, 16, 28, 36)	0.77	0.79	0.74
2. Tradition (9, 20, 25, 38)	0.81	0.71	0.69
3. Benevolence (12, 18, 27, 33)	0.9	0.92	0.52
4. Universalism (3, 8, 19, 23, 29, 40)	0.86	0.82	0.56
5. Self-direction (1, 11, 22, 34)	0.91	0.73	0.80
6. Stimulation (6, 15, 30)	0.72	0.63	0.84
7. Hedonism (10, 26, 37)	0.83	0.78	0.75
8. Achievement (4, 13, 24, 32)	0.86	0.88	0.85
9. Power (2, 17, 39)	0.53	0.82	0.55
10. Security (5, 14, 21, 31, 35)	0.80	0.78	0.68

Below is a summary of the factor analysis results of the Portrait Values Questionnaire (PVQ), as presented in this table.

## Results

The data were analyzed using several statistical procedures, which are described below. It was hypothesized that parent–child religious distance in religious values would be found to be statistically significant in both homogeneous Modern-Orthodox family groups and in heterogeneous Modern-Orthodox-secular family groups due to secularism.

This hypothesis was evaluated using two measurements of parental values in accordance with the differentiation process, suggesting that parents have two different sets of values. Parental personal religious values (values that parents themselves personally hold and respect) and parents' socialization of religious values (the values that parents want their children to adopt) compared to children's personal religious values.

It was also hypothesized that the effect sizes of statistically significant differences in the parent–child religious distance would be greater in heterogeneous Modern-Orthodox family groups than in homogeneous Modern-Orthodox family groups.

Lastly, it was hypothesized that parent–child religious distance would not vary between boys and girls due to modernism and postmodernism, where, according to research, parents aim to instill very high religious values in their children regardless of the child's gender. Table 6 shows the summary of the results of the factor analysis of the Portrait Values Questionnaire (PVQ) as presented in Table 5.

**Table 6:** Factor analysis of the portrait values questionnaire (PVQ): summary of eigenvalues and % of explained variance.

**Factor**	**1**	**2**	**3**	**4**	**5**	**6**	**7**	**8**	**9**	**10**
Eigenvalue	2.35	2.16	2.88	2.26	2.75	2.52	3.13	3.89	1.73	1.61
% Exp. Var.	5.86	5.15	7.20	5.65	6.89	6.31	7.82	9.73	4.33	4.03

### Parental personal religious values in comparison to parents' socialization of religious values

The repeated-measures analysis of variance yielded the following results:

A significant interaction was found between the interaction of group, differentiation process (parents' personal values and parents' socialization of values), and religious values [*F*_(2,139)_ = 5.6, *p* = 0.005, η^2^ = 0.08].

A follow-up *post hoc* analysis using the Bonferroni procedure was conducted with respect to the three-factor interaction between the variable “group,” “differentiation process,” and “religious values,” which indicated the following results.

In all family groups, parental socialization of values was higher than parental personal religious values. In the homogeneous Modern-Orthodox family group, parents' socialization of religious values (*M* = 4.94, *SD* = 0.64) was higher than parents' personal religious values (*M* = 4.7, *SD* = 0.72).

In the mixed “Modern-Orthodox father-secular mother” family group, parents' socialization of religious values (*M* = 4.8, *SD* = 0.116) was higher than parents' personal religious values (*M* = 4.3, *SD* = 0.108).

In the mixed “Modern-Orthodox mother-secular father” family group, parents' socialization of religious values (*M* = 4.5, *SD* = 0.108) was higher than parents' personal religious values (*M* = 4.20, *SD* = 0.101).

### The distance between parental values and children's own values

The repeated-measures analysis of variance yielded the following results.

A significant main effect of the variable group [*F*_(2,139)_ = 10.5, *p* < 0.01, η^2^ = 0.13] was found of “parent–child transmission” [(father's own values against the child's own values) or (mother's own values against the child' own values)], such that the Modern-Orthodox group (*M* = 4.6, *SD* = 0.06) was significantly higher in the transmission of religious values than the “Modern-Orthodox” father “secular” mother *(M* = 4.3, *SD* = 0.09) and the “Modern-Orthodox” mother and “secular” father (*M* = 4.2, *SD* = 0.08) groups.

No significant main effect was found of “parent–child transmission” [(father's own values against the child's own values) or (mother's own values against the child's own values)] with respect to group (“Modern-Orthodox father and mother,” “Modern-Orthodox father-secular mother” and “Modern-Orthodox mother-secular father”). No significant main effect was found between group, religious values, and “parent–child transmission.”

### The distance between parents' socialization of values and children's own values

The repeated-measures analysis of variance yielded the following results.

A significant main effect of the variable group [*F*_(2,139)_ = 7.4, *p* < 0.01, η^2^ = 0.09] was found of “parent–child transmission” [(father socialization of values against the child's own values) or (mother socialization of values against the child's own values)], such that the Modern-Orthodox group (*M* = 4.8, *SD* = 0.062) was significantly higher in the transmission of religious values than the heterogeneous “Modern-Orthodox” father and “secular” mother group (*M* = 4.39, *SD* = 0.09).

**Figure 1:**
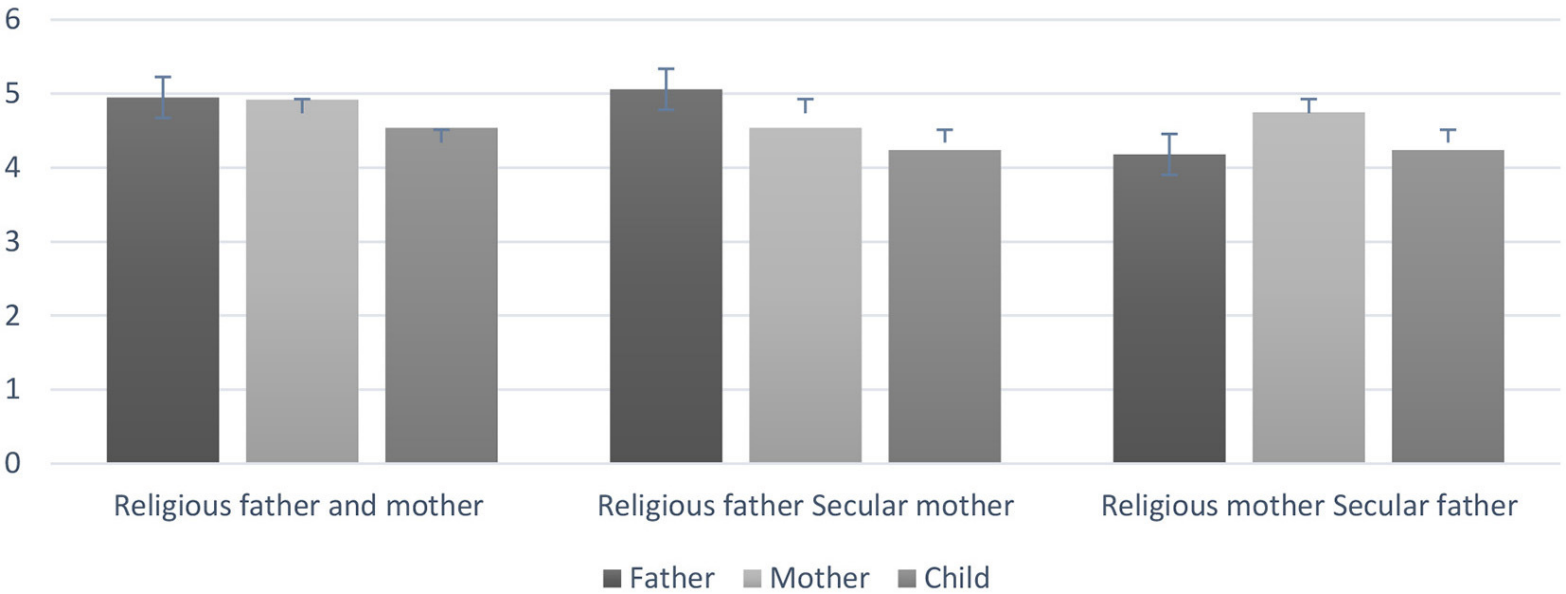
Means and standard deviations of the three groups of study, with respect to the religious distance in religious values between father and child and mother and child.

A significant main effect was found of “parent–child transmission” [(father social values against the child's own values) or [mother social values against the child's own values)] with respect to the “Modern-Orthodox father and mother, “Modern-Orthodox father-secular mother” and “Modern-Orthodox mother -secular father” groups [*F*_(2,138)_ = 22.81, *p* < 0.001, η^2^ = 0.14], where parents' socialization of values (*M* = 4.73, *SD* = 0.65) were significantly higher than the child's own values (*M* = 4.34, *SD* = 0.55) in all groups. There is a significant interaction between group, religious values, and “parent–child transmission” [*F*_(4,278)_ = 10.09, *p* < 0.001, η^2^ = 0.13] (see Table 7 and Figure 1).

**Table 7:** Means and standard deviations of the three groups of study, with respect to the religious distance in religious values between father and child and mother and child.

**Family type**	**Religious values**					
	**Father**		**Mother**		**Child**	
	***M***	***SD***	***M***	***SD***	***M***	***SD***
Modern-Orthodox father and mother	4.95	0.59	4.92	0.64	4.54	0.63
Modern-Orthodox father and Secular mother	5.06	0.66	4.54	0.64	4.24	0.55
Modern-Orthodox mother and Secular father	4.18	0.95	4.75	0.88	4.24	0.65

A follow-up post hoc analysis using the Bonferroni procedure was conducted with respect to the three-factor interaction between the variables “group,” “parent–child,” and “religious values,” which indicated the following results:

#### Religious values

In the homogeneous Modern-Orthodox “father and mother” family group, both the religious mother (*M* = 4.92, *SD* = 0.64) and the religious father (*M* = 4.95, *SD* = 0.59) were higher in the socialization of religious values than their child's own religious values (*M* = 4.54, *SD* = 0.63) (*p* < 0.001) (*d* = 0.63).

In the mixed “Modern-Orthodox father-secular mother” family group, the religious father (*M* = 5.06, *SD* = 0.66) was higher in the socialization of religious values when compared with his child's own religious values (*M* = 4.24, *SD* = 0.55) (*p* < 0.001) (*d* = 1.38).

In the mixed “Modern-Orthodox mother secular father” family group, the religious mother was significantly higher in the socialization of religious values (*M* = 4.75, *SD* = 0.88) in comparison to her child's own religious values (*M* = 4.24, *SD* = 0.65), (*p* < 0.001) (d = 0.65).

## Child's gender in the socialization of values from parents to children

The repeated measure analysis of variance yielded no significant difference in parent–child transmission of values in the three family groups due to gender [*F*_(4,272)_ = 2.2, ns, η^2^ = 0.003].

In summary, the results support the first hypothesis, indicating that there is a difference between parents' socialization of religious values and parents' personal religious values in all family groups. The results showed that parents' socialization of religious values is higher than parents' actual religious values, where parents' actual religious values were found to be statistically insignificant to children's personal religious values.

The results support the first hypothesis with respect to parents' socialization of religious values, showing that parents' socialization of religious values and children's personal values are statistically significant in both homogeneous Modern-Orthodox family groups and heterogeneous Modern-Orthodox-secular family groups.

In addition, the results partly support the second hypothesis, indicating that the effect sizes in the religious distance of religious values are larger for heterogeneous Modern-Orthodox-secular family groups than homogenous Modern-Orthodox family groups. The results show a greater effect size in the parent–child religious distance of religious values between the Modern-Orthodox father and his child in the heterogeneous family groups compared to the religious distance between the Modern-Orthodox father and his child in the homogeneous family type. However, effect sizes were similar with respect to the homogeneous Modern-Orthodox father and mother family group and the Modern-Orthodox mother and secular father family group.

Finally, the result supports the last hypothesis, arguing that there is no significant effect of gender on the socialization of religious values due to modernism and postmodernism, where, according to research, parents aim to instill very high religious values in their children regardless of the child's gender.

## Discussion

The findings of the study confirm the study's first hypothesis, indicating that there is a difference between parents' socialization of religious values and parents' personal religious values since parents' socialization of values and parental values are sometimes observed by the parents as two different sets of values (Benish-Weisman et al., 2013). “Sometimes parents see the need to differentiate between their personal values and what they want for their children” (Tam and Lee, 2010, p. 175), especially religious parents in fear of modernism.

The hypothesis was further confirmed with respect to parents' socialization of religious values, such that parent-child religious distance in religious values was found to be statistically significant in the parent–child socialization of religious values in both Modern-Orthodox-secular heterogeneous family groups and in heterogeneous Modern-Orthodox family groups. It was observed that adolescent children are significantly less religious than both their Modern-Orthodox mothers and fathers want them to be, and the effect sizes range from medium to high (Cohen, 1992).

The results show that the family type (homogeneous/mixed) does not predict the similarity or differences in religious values between children's and parents' socialization of values. However, the findings show that the similarity or differences between children's and parents' socialization of values are determined by the religious/secular identity of the parent and not whether the family type is homogeneous Modern-Orthodox or mixed Modern-Orthodox-secular family groups.

Previous research has recommended that parents and adolescent children are similar in their religiousness in religiously homogeneous family groups. However, in heterogeneous religious-secular family groups, previous research has shown that parent–child religious distance in values is likely to occur due to the child having to choose between two different sets of values.

This study supports the growing body of research that suggests that the (failed) transmission of religion from parents to children functions as a mechanism of secularization (Cragun, 2017).

Throughout history, there has been a conflict between secular and religious Jews. “The historical status quo, which arranged for workable relations between the religious and secular sections of the Jewish population in Israel, has been under constant assault since the 1970s and especially since the 1990s” (Katz, 2010, p. 4). “We are witnessing not merely an extrapolation of past animosities into present-day realities but rather important changes in the character, context, and dramatis personae of the religious-secular confrontation” (Cohen and Susser, 2000, p. 14).

What is the trigger at the root of the secular-religious conflict? How does it happen that one nation splits into two contrasting groups with different beliefs and ideas? Part of the answer lies in the clash over human values. “Values play a central role in a wide variety of conflicts” (Druckman et al., 1988, p. 489). According to Schwartz (2013), “values may play little role *[sic]* in behavior except when there is value conflict” (p. 121).

Struch and Schwartz (1989) used the term ‘intergroup bias': “This bias typically takes the form of in-group favoritism, a preference for one's in-group over the out-group” (p. 364).

The conflict in Israel between religiosity and secularity is also manifested as an inner conflict in Modern-Orthodox parents' socialization of values, especially due to the geographical proximity to the traditional and secular family groups, wherein, in a heterogeneous family group, this proximity exists both inside the family as well as outside the family.

The Modern-Orthodox parent understands the threat of secularism to their child's religious values, and therefore, they aim to instill very high religious values in them, which will be able to counteract the in-group secular values.

However, according to current research, the religious partners could not transmit the high level of religious values that they wanted to pass down to their children. The religious values that the religious parent wanted to instill in their child were on a higher level than the actual religious values of the child.

Erik Erikson was the first theorist to coin the term *identity crisis*. Erikson believed that the identity crisis was one of the most important conflicts that people face during the developmental process. According to Erikson, an identity crisis is a time of intensive analysis and exploration of diverse ways of looking at oneself (Frisch-Atias, 2012).

Canadian developmental psychologist James Marcia was one of the most important theoreticians to develop Erikson's theory of identity crisis. Marcia primarily focused on adolescent development when addressing Erikson's notion of an identity crisis. Marcia argued that two distinct parts are involved in the identity crisis: the first consists of reexamining one's values and choices and the second is the commitment to those values and choices. Marcia posited that the adolescent stage consists of the degree to which one is committed to an identity. One such identity is religion (Hadad, 2009). A crisis of values is caused by modernism and postmodernism in religious identity formation.

When a religious child grows up in the modern and postmodern world, there might be a clash between the religious values that they are taught at home to follow and the more universal values and lifestyles in the modern and postmodern world, and as such, a religious identity crisis might be caused in adolescents (Cohen-Malayev, 2008; Steinberg, 2008; Frisch-Atias, 2012).

The case may also be that religious parents themselves knowingly attempt to transmit a very high degree of religious values because they want to protect the child from the religious identity crisis that might be caused by the secular values of the modern and postmodern world. However, the results show that religious parents' aim of instilling such a high degree of religious values in their children is not fully accomplished. A recent study found that teenage children are 12% less religious than their fathers and 17% less religious than their mothers (Cragun et al., 2018). These studies do not suggest that children growing up in religious households are not religious but that their religious values are not as strong as the values their parents wished to instill in them.

The results also partly support the second hypothesis, indicating that parent–child religious distance in religious values is greater in the value transmission of heterogeneous Modern-Orthodox-secular family groups in comparison with homogeneous Modern-Orthodox family groups due to a double conflict over values both in the family environment and in the secular trends of modernism and postmodernism. As such, the effect sizes of statistically significant results are greater in a parent–child religious distance of religious values in heterogeneous Modern-Orthodox family groups in comparison to homogeneous Modern-Orthodox family groups.

The results show that, when comparing the parent–child religious distance in religious values between the homogeneous Modern-Orthodox family groups and the heterogeneous “Modern-Orthodox” father “secular” mother family groups, the hypothesis was confirmed. However, when comparing the parent–child religious distance in religious values between the homogeneous Modern-Orthodox family groups and the heterogeneous Modern-Orthodox mother secular father family groups, the hypothesis was rejected.

A possible answer might lie in the way that religious values are portrayed in the Jewish religion. The Jewish tradition differentiates between the religious obligations of men and women, placing greater responsibility on men to fulfill religious duties (Loewenthal et al., 2002). It could be because of the differences in religious obligation between men and women in the Jewish religion that Jewish men might feel that they have a greater responsibility in transmitting religious beliefs and religious values to their children. As such, they attempted to transmit an exceedingly high degree of religious values because they want to protect the child from the religious identity crisis that might be caused by the secular values of the modern and postmodern worlds. The findings of the study further support our third hypothesis, indicating that there are significant differences between religious parents and their children in the socialization of religious values by both girls and boys, since the emerging adulthood stage of development produces, knowingly or unknowingly, a conflict in their child's values, regardless of gender, between the values that the modern and postmodern world has to offer and the religious values that the religious parent wishes to transmit (Ben-Rafael and Ben-Chaim, 2006; Cohen-Malayev, 2008).

Finally, the results confirm the third hypothesis, indicating that parent–child religious distance does not vary for boys or girls due to modernism and postmodernism, where, according to research, parents aim to instill very high religious values in their children regardless of the child's gender.

## Conclusion

This study is important in that it improves our understanding of how culture and religion influence the parent–child value transmission as well as the extent of religious distance in values between parents and children.

The uniqueness of this study is in the fact that it further supports the current literature that suggests that parent–child religious distance in religious values is not a product of the type of family that the child grows up in (heterogeneous or homogeneous). However, the religious distance in religious values was also found in families where both parents are in agreement over religious values due to modernism and postmodernism and new trends in secularity.

The study has several limitations. First, there is a need to reconfirm the findings of this study with larger samples. Second, only one sibling per family was included in the study. Further research is needed to test and strengthen the results of the study by studying several siblings in the same family with respect to the relationship between values and religiosity/secularity. Third, this research is not longitudinal in design, and incorporating a longitudinal approach would have greatly contributed to the results of the study.

## Data availability statement

The datasets presented in this article are not readily available because they are subject to ongoing research. Requests to access the datasets should be directed to elaluria@yahoo.com.

## Ethics statement

The studies involving human participants were reviewed and approved by Bar Ilan University, Israel. The patients/participants provided their written informed consent to participate in this study.

## Author contributions

EL conceived, planned, and carried out the experiment and also wrote the manuscript.

## References

[B1] Aldous J. Hill R. (1965). Social cohesion, lineage type, and intergenerational transmission. Soc. Force 43, 471–482. 10.2307/2574453

[B2] Arnett J. J. (2000). Emerging adulthood: a theory of development from the late teens through the twenties. Am. Psychol. 55, 469–480. 10.1037/0003-066X.55.5.469 10842426

[B3] Axinn W. G. Thornton A. (1993). Mothers, children and cohabitation: The intergenerational effects of attitudes and behavior. Am. Sociol. Rev. 58, 233–246.

[B4] Bader C. D. Desmond S. A. (2006). Do as I say and as I do: The effects of consistent parental beliefs and behaviors upon religious transmission. Sociol. Reli. 67, 313–329. 10.1093/socrel/67.3.313

[B5] Barni D. Ranieri S. Scabini E. Rosnati R. (2011). Value transmission in the family: do adolescents accept the values their parents want to transmit? J. Moral Educ. 40, 105–121. 10.1080/03057240.2011.553797

[B6] Benish-Weisman M. Levy S. Knafo A. (2013). Parents differentiate between their personal values and their socialization values: The role of adolescents' values. J. Res. Adolesc. 23, 614–620. 10.1111/jora.12058

[B7] Ben-Rafael E. Ben-Chaim L. (2006). Jewish Identities in An Era of Multiple Modernities. Tel Aviv: The Open University.

[B8] Bisin A. Verdier T. (2010). The economics of cultural transmission and socialization (NBER Working Paper No. 16512). Cambridge, MA: National Bureau of Economic Research.

[B9] Carlson D. L. Knoester C. (2011). Family structure and the intergenerational transmission of gender ideology. J. Fam. Issue. 32, 709–734. 10.1177/0192513X10396662

[B10] Clark C. A. Worthington E. L. Danser D. B. (1988). The transmission of religious beliefs and practices from parents to firstborn early adolescent sons. J. Marriage Fam. 50, 463–472.

[B11] Cohen A. B. Susser B. (2000). Israel and the Politics of Jewish Identity: The Secular-Religious Impasse. Baltimore, MD: Johns Hopkins University Press.

[B12] Cohen J. (1992). A power primer. Psychol. Bullet. 112, 155–159. 10.1037/0033-2909.112.1.155 19565683

[B13] Cohen-Malayev M. (2008). Religious exploration and identity: The tension between religion and religiosity - Examination of the internalization process of Jewish religious values in modern orthodox context (Unpublished doctoral dissertation). Ben-Gurion University, Be'er Sheva.

[B14] Copen C. E. Silverstein M. (2008). The transmission of religious beliefs across generations: do grandparents matter? J. Comp. Family Studies 39, 59–71. 10.3138/jcfs.39.1.59

[B15] Cragun R. T. (2017). The Declining Significance of Religion: Secularization in Ireland. In M. J. Breen (Ed.), Values and Identities in Europe: Evidence from the European Social Survey. Oxford, England: Routledge, 17–35.

[B16] Cragun R. T. Hammer J. H. Nielsen M. Autz N. (2018). Religious/secular distance: how far apart are teenagers and their parents? Psychol. Relig. Spirituality 10, 288. 10.1037/rel0000205

[B17] Crockett A. Voas D. (2006). Generations of decline: religious change in 20th-century Britain. J. Sci. Study Relig. 45, 567–584. 10.1111/j.1468-5906.2006.00328.x

[B18] Cunningham M. (2001). Parental influences on the gendered division of house work. Am. Sociol. Rev. 66, 184–203. 10.2307/2657414

[B19] Druckman D. Broome B. J. Korper S. H. (1988). Value differences and conflict resolution: Facilitation or delinking? J. Conflict Resolut. 32, 489–510. 10.1177/0022002788032003005

[B20] Even-Zohar A. (2015). Grandparent-grandchild relationships in Israel: A comparison between different Jewish religious groups. J. Intergenerat. Relationship. 13, 75–88. 10.1080/15350770.2015.992876

[B21] Flor D. L. Knapp N. F. (2001). Transmission and transaction: Predicting adolescents' internalization of parental religious values. J. Family Psychol. 15, 627–645. 10.1037/0893-3200.15.4.627 11770469 PMC8786438

[B22] Fontaine J. R. J. Duriez B. Luyten P. Corveleyn J. Hutsebaut D. (2005). Consequences of a multidimensional approach to religion for the relationship between religiosity and value priorities. In. J. Psychol. Relig. 15, 123–143. 10.1207/s15327582ijpr1502_2

[B23] Frisch-Atias E. (2012). Parental influence during religious identity formation adolescent's perspective (Unpublished master's dissertation). School of Education, Bar-Ilan University, Ramat Gan.

[B24] Glenn N. D. Kramer K. B. (1987). The marriages and divorces of the children of divorce. J. Marriage Fam. 49, 811–825.

[B25] Gniewosz B. Noack P. (2012). Mamakind or papakind? [Mom's child or Dad's child]: Parent-specific patterns in early adolescents' intergenerational academic value transmission. Learn. Individ. Diff. 22, 544–548. 10.1016/j.lindif.2012.03.003

[B26] Hadad T. (2009). The “religiously-lite”: A model for religious Identity in the postmodern era (Unpublished master's dissertation). School of Education, Bar-Ilan University, Ramat Gan.

[B27] Hayes B. C. Pittelkow Y. (1993). Religious belief, transmission, and the family: An Australian study. J. Marriage Fam. 55, 755–759.

[B28] Hofmann-Towfigh N. (2007). Do students' values change in different types of schools? J. Moral Educ. 36, 453–473. 10.1080/03057240701688010

[B29] Kapinus C. A. (2004). The effect of parents' attitudes toward divorce on offspring's attitudes: Gender and parental divorce as mediating factors. J. Fam. Issue. 25, 112–135.

[B30] Katz Y. J. (2010). The state approach to Jewish and non-Jewish education in Israel. Compar. Educ. 46, 325–338. 10.1080/03050068.2010.503741

[B31] Katz Y. J. Schmida M. (1992). Validation of the student religiosity questionnaire. Educ. Psychol. Measurement 52, 353–356. 10.1177/0013164492052002010

[B32] Knafo A. Galansky N. (2008). The influence of children on their parents' values. Soc. Pers. Psychol. Compass. 2, 1143–1161.

[B33] Knafo A. Schwartz S. H. (2003). Parenting and adolescents' accuracy in perceiving parental values. Child Dev. 74, 595–611. 10.1111/1467-8624.7402018 12705575

[B34] Kohn M. L. Slomczynski K. M. Schoenbach C. (1986). Social stratification and the transmission of values in the family: A cross-national assessment. Sociol. Forum. 1, 73–102. 10.1007/BF01115074

[B35] Lazar A. Kravetz S. Frederich-Kedem P. (2002). The multidimensionality of motivation for Jewish religious behavior: Content, structure, and relationship to religious identity. J. Scient. Study Relig. 41, 509–519. 10.1111/1468-5906.00134

[B36] Lee R. L. M. (2006). Reinventing modernity: reflexive modernization vs. liquid modernity vs. multiple modernities. Eur. J. Soc. Theory 9, 355–368. 10.1177/1368431006065717

[B37] Loewenthal K. M. MacLeod A. K. Cinnirella M. (2002). Are women more religious than men? Gender differences in religious activity among different religious groups in the UK. Person Ind. Diff. 32, 133–139. 10.1016/S0191-8869(01)00011-3

[B38] Min J. Silverstein M. Lendon J. P. (2012). Intergenerational transmission of values over the family life course. Adv. Life Course Res. 17, 112–120. 10.1016/j.alcr.2012.05.001

[B39] Myers S. M. (2004). Religion and intergenerational assistance: distinct differences by adult children's gender and parents' marital status. Sociol. Q. 45, 67–89. 2004.tb02398.x 10.1111/j.1533-8525.2004.tb02398.x

[B40] Nelson M. R. Otnes C. C. (2002). Exploring cross-cultural ambivalence: anthography of intercultural wedding message boards. J. Bus. Res. 58, 89–95. 10.1016/S0148-2963(02)00477-0

[B41] O'Bryan M. Fishbein H. D. Ritchey P. N. (2004). Intergenerational transmission of prejudice, sex role stereotyping, and intolerance. Adolescence. 39, 407–426. 15673220

[B42] Okun S. Nimrod G. (2017). Online ultra-orthodox religious communities as a third space: A netnographic study. Int. J. Commun. 11, 2825–2841.

[B43] Ozorak E. W. (1989). Social and cognitive influences on the development of religious beliefs and commitment in adolescence. J. Sci Study Religion 28, 448–463. 10.2307/1386576

[B44] Phalet K. Schonpflug U. (2001). Intergenerational transmission in Turkish immigrant families: Parental collectivism, achievement values and gender differences. J. Compar. Fam. Stud. 32, 489–504.

[B45] Popenoe D. (1993). American family decline: 1960-1990: A review and appraisal. J. Marriage Family 55, 527–542. 10.2307/353333

[B46] Roccas S. (2005). Religion and value systems. J. Soc. Issue. 61, 747–759. 10.1111/j.1540-4560.2005.00430.x

[B47] Rokeach M. (1973). The Nature of Human Values. New York: Free.

[B48] Saroglou V. Delpierre V. Dernelle R. (2004). Values and religiosity: A meta-analysis of studies using Schwartz's model. Pers. Individ. Diff. 37, 721–734. 10.1016/j.paid.2003.10.005

[B49] Saroglou V. Munoz-Garcia A. (2008). Individual differences in religion and spirituality: an issue of personality traits and/or values. J. Sci. Study Religion. 47, 83–101. 00393.x 10.1111/j.1468-5906.2008.00393.x

[B50] Schonpflug U. (2001). Introduction: cultural transmission - a multidisciplinary research field. J. Cross-Cultural Psychol. 32, 131–134. 10.1177/0022022101032002001

[B51] Schwadel P. (2010). Period and cohort effects on religious nonaffiliation and religious disaffiliation: a research note. J. Sci. Study Religion 49, 311–319. 10.1111/j.1468-5906.2010.01511.x

[B52] Schwartz S. H. (1992). Universals in the content and structure of values: theoretical advances and empirical tests in 20 countries. Advances in Exp. Soc. Psychol. 25, 1–65. 10.1016/S0065-2601(08)60281-6

[B53] Schwartz S. H. (2006). A theory of cultural value orientations: Explication and applications. Comp. Sociol. 5, 137–182. 10.1163/156913306778667357

[B54] Schwartz S. H. (2012). An overview of the Schwartz theory of basic values. Online Read. Psychol. Cult. 2, 1–20. 10.9707/2307-0919.1116

[B55] Schwartz S. H. (2013). Value priorities and behavior: Applying a theory of integrated value systems, in The Psychology of Values: The Ontario Symposium, Vol. 8 (Psychology Press).

[B56] Schwartz S. H. Huismans S. (1995). Value priorities and religiosity in four Western religions. Soc. Psychol. Q. 58, 88–107. 10.2307/2787148

[B57] Steinberg P. (2008). From conflict to resolution: Achieving cohesion in Jewish Identity among women from sub-culture groups in Israeli society (Unpublished doctoral dissertation). School of education, Bar-Ilan University, Ramat Gan.

[B58] Struch N. Schwartz S. H. (1989). Intergroup aggression: Predictors and distinctiveness from in-group bias. J. Pers. Soc. Psychol. 56, 364–373. 10.1037/0022-3514.56.3.364 2926634

[B59] Tam K. P. Lee S. (2010). What values do parents want to socialize in their children? The role of perceived normative values. J. Cross-Cult. Psychol. 41, 175–181. 10.1177/0022022109354379

[B60] Tam K. P. Lee S. L. Kim Y. H. Li Y. Chao M. M. (2012). Intersubjective model of value transmission: Parents using perceived norms as reference when socializing children. Pers. Soc. Psychol. Bull. 38, 1041–1052. 22539215 10.1177/0146167212443896

[B61] Wald K. D. Shye S. (1995). Religious influence in electoral behavior: The role of institutional and social forces in Israel. J. Polit. 57, 495–507.

[B62] World Population Review (2020). Most Educated Countries 2020. Available online at: https://worldpopulationreview.com/country-rankings/most-educated-countries

